# Development of Hybrid Electrospun Nanofibers: Improving Effects of Cellulose Nanofibers (CNFs) on Electrospinnability of Gelatin

**DOI:** 10.3390/foods13132114

**Published:** 2024-07-02

**Authors:** Farnaz Hajieghrary, Babak Ghanbarzadeh, Akram Pezeshki, Saeed Dadashi, Pasquale M. Falcone

**Affiliations:** 1 Department of Food Science and Engineering, Faculty of Agriculture, University of Tabriz, Tabriz 5166616471, Iranakram.pezeshki@tabrizu.ac.ir (A.P.); dadashis@tabrizu.ac.ir (S.D.); 2 Department of Agricultural, Food, and Environmental Sciences, University Polytechnical of Marche, Brecce Bianche 10, 60131 Ancona, Italy

**Keywords:** CNF, electrospinning, hybrid nanofiber, gelatin, fiber diameter

## Abstract

Cellulose nanofibers (CNFs) were used to improve the electrospinnability of the gelatin protein in a water/ethanol/acetic acid (3:2:3, *v*/*v*) solution. The effects of different concentrations of CNFs (0.5–4%) on the important physical properties of the gelatin solution (15%), including rheology, conductivity, and surface tension, were investigated. The apparent viscosity and shear-thinning behavior were increased by increasing the CNF concentration from 0 to 4% at a low shear rate (<10 s^−1^). CNFs also increased the electrical conductivity and surface tension of the gelatin solution. Scanning electron microscopy (SEM) images revealed uniformly ordered structures with good continuity without fracture or bead formation in all hybrid nanofibers. They also showed that the average diameters of fibers decreased from 216 nm in the pure gelatin nanofibers to 175.39 nm in the hybrid gelatin/CNF (4%) ones. Differential scanning calorimetry (DSC) results showed that CNFs increased T_g_, and X-ray diffraction (XRD) analysis showed that the electrospinning process caused the formation of more amorphous structures in the gelatin/CNF hybrid nanofibers. The tensile test indicated that by adding 2% CNFs, the ultimate tensile strength (UTS) and strain at break (SB) of nanofiber mats increased from 4.26 to 10.5 MPa and 3.3% to 6.25%, respectively. The current study indicated that incorporating CNFs at the optimal concentration into a gelatin solution can improve the resulting hybrid nanofibers’ morphology, average diameter, and mechanical properties.

## 1. Introduction

Electrospinning is an uncomplicated, adaptable, and economical method used to create films made of sub-micron or nanoscale fibers from biopolymer or synthetic polymer solutions or melts [1]. This process is achieved by applying a high voltage between a syringe containing the biopolymer solution/melt and a collector plate. The electric field causes the biopolymer solution drop to form into a Taylor cone and then a jet stream that is stretched and thinned by electrostatic forces due to the presence of chain entanglements and its elongational viscosity. Finally, the solvent is evaporated from the jet, leaving solid biopolymer fibers that are deposited on the collector plate, forming a film or mat. The electrospun nanofiber film is a thin layer of aligned nanofibers that form a smooth surface, while an electrospun nanofiber mat is a collection of randomly oriented nanofibers that form a non-woven fabric.

To resist stretching and bending motions during electrospinning, it is crucial to have a high concentration of the polymer. This ensures sufficient overlap between the molecules, leading to the formation of an entangled network. Electrospun nanofibers have gained significant attention for their application in food packaging and encapsulation technologies in the past few years, such as in (1) the improvement of mechanical and optical properties, barrier performance, and thermal stability by using electrospun nanofibers as intermediate layers or coatings and fillers in packaging materials and (2) the development of active and intelligent packaging films with the use of active compounds or sensor/indicator-loaded nanofibers in packaging materials [2].

In contrast to traditional casting films, electrospun nanofibers possess several distinct characteristics. These include a high surface-to-volume ratio, a nanoporous structure, high porosity, and a high absorbance capacity [3]. These properties make electrospun nanofibers more sensitive to changes in acidity/alkalinity in their environment and allow for better control over the release of bioactive compounds that are incorporated within them. The electrospinning ability of biopolymers and the properties of electrospun films/mats can be tuned by modifying the biopolymer (type, concentration, and molecular weight), solvent (surface tension, dielectric constant, and electrical conductance), or process parameters (such as voltage, flow rate, syringe–collector distance, and humidity) or by using additives such as cross-linkers and surfactants [4].

The electrospinning of proteins is difficult because of several challenges: (1) some proteins (especially plant proteins) tend to form globular structures (tertiary and quaternary structures) through different types of bonds that are hard to stretch and align into fibers; (2) the polyelectrolytic nature of the polypeptide chains prevents the effective entanglement of chains and the formation of the jet stream; and (3) the sensitivity of proteins to environmental conditions, such as the temperature, pH, and solvent, which can cause the denaturation, aggregation, or degradation of the proteins during electrospinning [4,5].

Gelatin is a well-known and widely used animal protein that has higher electrospinnability than other proteins for the following reasons: (1) gelatin is derived from collagen, which is a fibrous protein that has a triple-helical structure that can be easily stretched and aligned into fibers by electrostatic forces; (2) gelatin can dissolve in various solvents, such as water, acetic acid, formic acid, and ethanol, due to having a high content of polar amino acids, such as serine, tyrosine, and aspartic acid, which can form hydrogen bonds with the solvent molecules; (3) it forms viscous solutions that enhance the entanglement and electrospinnability of the solution; and (4) gelatin has a high content of proline and hydroxyproline, which are cyclic amino acids that can stabilize the helical structure and fiber formation [6,7,8].

Gelatin-based films have found commercial applications in the food industry as wrapping materials to preserve food quality and extend shelf life, particularly in the meat and fishery sectors [9,10]. Gelatin has been utilized as an electrospinning film for the creation of active packaging [11]. Cetinkaya et al. (2024) conducted a study on the use of electrospun gelatin nanofibers as an intelligent packaging layer for Hake fish (*Merluccius Merluccius*) fillets. The nanofibers were enriched with black elderberry extract, Au nanoparticles, and SnO_2_ [12]. Lin et al. (2018) produced active gelatin nanofiber mats containing glycerin and ε-Poly-lysine, which exhibited remarkable antibacterial properties against *Listeria monocytogenes* and were effective in preserving beef [13]. In other research, Zhou et al. (2020) fabricated electrospun gelatin nanofibers containing angelica essential oil for food packaging purposes [14].

Gelatin-based packaging lacks adequate mechanical strength, water resistance, and desired bioactivities, such as antibacterial and antioxidant properties, which are important for active food packaging [15]. A recent approach to addressing these limitations involves the use of nanomaterials, which can enhance the strength of biopolymer-based films through the formation of nanocomposites [16]. Bionanocomposites represent a novel concept in which materials are combined and applied by incorporating nanoscale bioparticles as reinforcement (filler) [17]. When dispersing nanometer-sized particles within the biopolymer matrix, these nanocomposites demonstrate superior mechanical, thermal, optical, physiochemical, and barrier properties compared to pure polymers [18].

Cellulose materials at the nanometer scale have become a significant area of research. Cellulose nanoparticles are primarily derived from plants through mechanical and chemical processes, or a combination of both [19]. Cellulose nanofibers (CNFs) are composed of nanosized cellulose fibrils with a high aspect ratio, meaning their length is much greater than their width. These fibrils typically have widths ranging from 5 to 20 nanometers and lengths that can reach several micrometers. CNFs are isolated from any cellulose-containing source by mechanical treatments, such as ball milling, high-pressure homogenization, grinding or microfluidization, and ultrasonic techniques. CNFs have lower crystallinity and rigidity and higher fiber lengths, aspect ratios, and flexibility than cellulose nanocrystals (CNCs), which are highly crystalline nano-cellulose produced by acid hydrolysis of cellulose fibers and the deletion of amorphous regions in cellulose [20,21,22,23].

CNFs can form nanocomposite polymers or hybrid polymers with biopolymers, in which they act as a nanofiller or a second polymer in the electrospinning of biopolymers, respectively. In the nanocomposite polymer, the individual constituents (polymers and CNFs) retain their separate identities within the composite but work together to enhance functional properties (spinnability, mechanical, thermal, and barrier properties). Unlike a composite polymer, in a hybrid polymer, the CNF is blended with the polymer at the molecular level as a second polymer to create a new compound, and the individual constituents are not distinguishable from each other. This hybrid polymer can exhibit synergistic effects and even new functionalities that are not necessarily found in the individual components. As a nanofiller, CNFs can be added to a biopolymer solution to form a composite and improve the nanofibers, as well as to enhance the stability of the solution. As a second polymer, CNFs can be blended with another biopolymer and form hybrid nanofibers.

Cellulose nanofibers (CNFs) are utilized as matrix-reinforcing elements to enhance thermo-mechanical properties, reduce the water sensitivity of polymers, and maintain biodegradability. Consequently, CNFs have been assessed for their ability to reinforce various polymeric matrices in casted films using starch [24], carboxymethyl cellulose (CMC) [22], polyvinyl alcohol [25], agar [23], poly (lactic acid) [20], and other polymers [26]. The interest in utilizing CNFs has experienced a significant surge, leading to extensive research on its potential applications [27]. According to our knowledge, little work has been carried out on CNF–biopolymer-based electrospun nanofibers.

This research aimed to evaluate the potential improving effect of CNFs (as a nanofiller or second biopolymer) on the physicochemical properties and morphology of a gelatin-based electrospinning solution and electrospun fiber films/mats for application in active packaging. The obtained electrospun hybrid nanofibers with the optimal diameter and morphology were then evaluated by DSC, XRD, and tensile tests.

## 2. Material and Methods

### 2.1. Materials

Gelatin (220 bloom) was purchased from Halavat Gel (Tabriz, Iran). Cellulose nanofibers in the form of gel (3%), purity 99%≤, obtained from softwood by mechanical synthesis were obtained from Nano Novin Polymer Co., (Mazandaran, Iran). Acetic acid and ethanol of analytical grade were purchased from Merck Co., (Darmstadt, Germany). Distilled water was used in all the experiments.

### 2.2. Preparation of Electrospinning Solution

Gelatin (10, 15, and 20%, *w*/*v*) was dissolved in a water/ethanol/acetic acid mixture (3:2:3, *v*/*v*), and the solution was stirred at 40 °C for 2 h until the gelatin was completely dissolved. It was used as a control sample. For the samples with CNFs, the first cellulose nanofibers (0.5, 1, 2, and 4% based on polymer) were dispersed in a water/ethanol/acetic acid mixture (3:2:3, *v*/*v*) and sonicated for 20 min in an ultrasonic bath. The ultrasonication provided the necessary mechanical agitation to disperse the nanofibers. Subsequently, gelatin was added to the solution and stirred at 40 °C for 2 h until completely dissolved. The composite membranes were electrospun from the solutions without and with CNFs, labeled as CNF0, CNF0.5, CNF1, CNF2, and CNF4.

### 2.3. Electrospinning Conditions

The electrospinning processes were conducted using a homemade electrospinning machine at room temperature. The solution was loaded into a syringe and then placed in a syringe pump, which was horizontally aligned with the drum collector. Electrospinning was conducted with an applied voltage of 20 kV, a distance of 15 cm between the needle tip and collector, and a pump flow rate of 0.3 mL/h. The resulting electrospun nanofibers were collected on aluminum foil, which was placed on the rotating collector.

### 2.4. Measurement and Characterization

#### 2.4.1. Characterization of Electrospinning Solutions

##### Rheological Properties Measurement

The rheological behavior of spinning solutions created at different CNF ratios was analyzed using steady-shear tests. The measurement of the solution viscosity was performed using an Anton Paar rheometer, smart pave (Anton Paar GmbH, 102e, Graz, Austria), at 25 °C using the plate–plate sensor configuration (dia 25 mm, gap 1 mm). The range of the shear rate was between 0.1 1/s and 100 1/s. The collected data were then used to fit the power-law model (Equation (1)):

(1) $$\tau =k\cdot {\dot{\gamma }}^{n}$$

The variables τ, k, $\dot{\gamma }$, and n represent the shear stress (Pa), consistency coefficient (Pa.sn), shear rate (1/s), and flow behavior index (dimensionless), respectively.

##### Electrical Conductivity and Surface Tension Measurement

The electrical conductivities of gelatin solutions with and without CNFs were measured using a conductometer (AZ, model 8301, Taichung, Taiwan) at room temperature in triplicate. The surface tensions of solutions were measured using a tensiometer (Kruss GmbH, K100, Hamburg, Germany) by the Wilhelmy plate method at room temperature in three replications.

#### 2.4.2. Morphological Analysis

A field-emission scanning electron microscope (FESEM) (JSM-6700F; JEOL, Tokyo, Japan) was used to analyze the morphology of samples. The Digimizer software program (version 5.4.5, MedCalc Software Ltd., Ostend, Belgium) was utilized to calculate the average fiber diameter, distribution, and standard deviation by measuring 50 randomly selected nanofibers from each FE-SEM image based on the pixel intensity and contrast of the images.

#### 2.4.3. Fourier Transform Infrared Spectroscopy

The gelatin, CNFs, and gelatin/CNF hybrid nanofibers were analyzed using FTIR spectroscopy. The analysis was conducted using a spectrophotometer (Tensor 27 spectrometer; Bruker Co., Ettlingen, Germany) in the wavenumber range of 500–4000 1/cm. To prepare the samples, the nanofiber mats were ground into powder and mixed with KBr salt. The mixture was then compressed into small pellets approximately 1 mm thick, following the KBr-pellet method.

#### 2.4.4. Thermal Analysis

The thermal analysis of the nanofiber mats was performed using a differential scanning calorimeter (model DSC400/Cl, Sanaf, Tehran, Iran). A 6–7 mg nanofiber sample was cut into small pieces and placed in an aluminum pan. An empty pan was used as a reference. The thermal properties of the different mats were measured from 25 to 270 °C with a heating rate of 10 °C/min.

#### 2.4.5. X-ray Diffraction Analysis

The distribution and crystallinity of cellulose nanofibers within the composite film matrix were analyzed using X-ray diffraction (XRD). A Cu Kα radiation beam (λ = 1.54184 nm) was employed with a diffraction angle range of 5° < 2θ < 70°. The XRD analysis was conducted at a voltage of 30 kV and 20 mA using a Tongda TD 3700 X-ray machine (Dandong, China). The scan rate was set at 0.5 1/s with a step size of 0.02. All measurements were performed at room temperature.

#### 2.4.6. Mechanical Property Measurements

The ultimate tensile strength (UTS) and percentage of strain at break (SB) of the nanocomposite film were determined using a Sanaf Universal Testing Machine (Tehran, Iran) equipped with a 25 N load cell. The cells were loaded according to the ASTM Standard D882-01 protocol for thin membranes [28]. To measure the tensile strength, strip-shaped samples were prepared with dimensions of 0.01 mm thickness, 30 mm length, and 10 mm width. The stretching process was conducted at a rate of 5 mm/min. Each specimen was tested at least three times, and the average result is reported.

#### 2.4.7. Statistical Analysis

We conducted three replicates for all assays. Statistical analysis was performed using IBM SPSS Statistics 22 (IBM Corporation, Armonk, NY, USA) with a one-way analysis of variance (ANOVA). To identify significant differences among values, Duncan’s multiple test range (*p* < 0.05) was employed. The data are presented as the means of three replications with their respective standard deviations.

## 3. Results and Discussion

### 3.1. Physical Properties of Solutions

#### 3.1.1. Rheological Property Measurements

**Figure 1:**
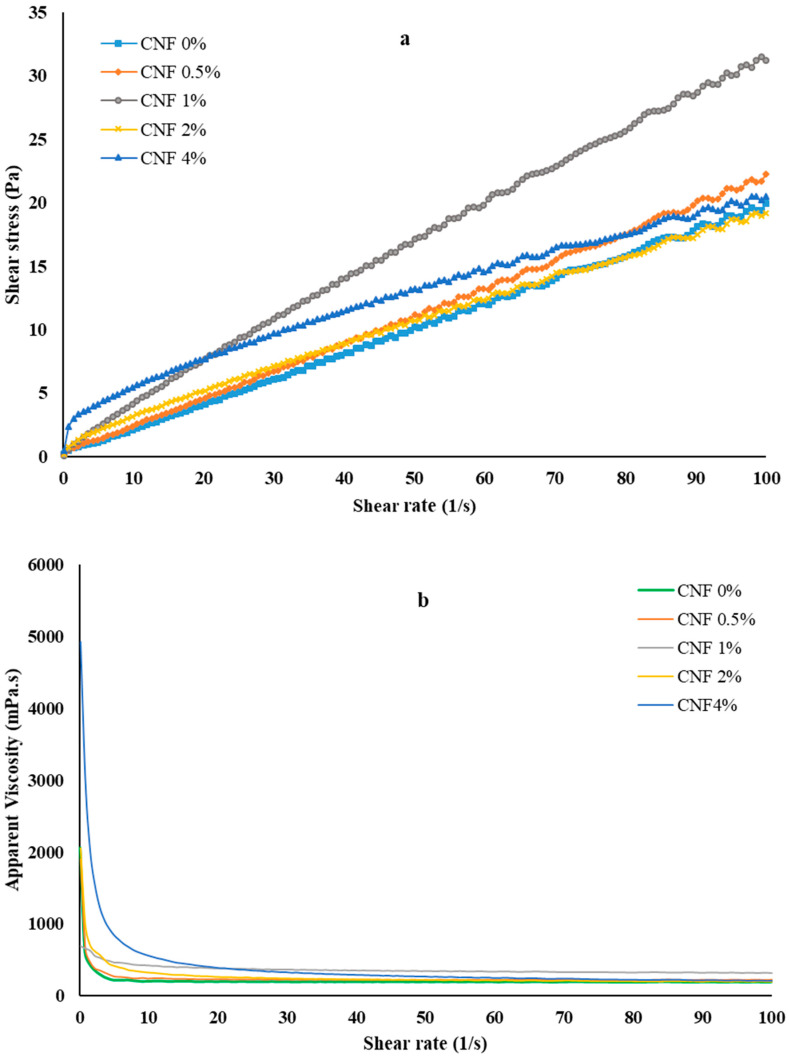
Flow curves of gelatin/CNF solutions used in the electrospinning process. (**a**) Shear stress–shear rate; (**b**) apparent viscosity–shear rate.

The rheological properties of gelatin solutions, consisting of 15% *w*/*v* gelatin dissolved in a mixture of water/ethanol/acetic acid (3:2:3, *v*/*v*), were determined using steady-shear rheometry in a shear rate range of 0.1–100 1/s (Figure 1a,b). All biopolymer solution samples exhibited shear-thinning (pseudoplastic) behavior, indicating that their resistance to flow decreases as the shear rate increases. The apparent viscosity was increased from 225.36 to 776.21 mPa·s by increasing the CNF concentration at a low shear rate (<10 1/s); however, it was not affected by the CNF concentration at a high shear rate.

In general, a spinning solution or melt should have a suitable apparent viscosity to achieve good electrospinnability. The viscosity should be high enough to prevent the jet from breaking up into droplets, but too high a viscosity may hinder jet elongation, the attenuation of electrical charge, and even the blockage of the syringe, which, in turn, prevents fiber formation [29]. On the other hand, an increase in viscosity causes an increase in the bead size and fiber diameters. According to previous reports, a spinning solution with a viscosity of 1–20 poise values is considered to be a solution suitable for electrospinning. However, these values may vary depending on the type and composition of the polymer solution or melt and the desired properties and applications of the nanofibers [30].

The power-law model is a mathematical equation that describes the relationship between the shear stress and the shear rate of a non-Newtonian fluid, such as a polymer solution or melt. The shear-stress–shear-rate data were accurately fitted (R^2^ > 0.9) using the power-law model, and the model parameters for different gelatin/CNF solutions are presented in Table 1. By considering flow index data (n), the pseudoelasticity of the solutions was increased by increasing the CNF concentration from 0.5 to 4%. A lower value of n generally indicates a higher entanglement of molecules, which is crucial for achieving nanofibers through electrospinning [31].

The consistency index (k value) reflects the degree of thickening of the polymer solution, which is correlated with viscosity. A higher k value signifies a greater entanglement/interaction between CNFs and gelatin at the molecular level. The polymer solution containing 4% CNFs exhibited the highest k value and the lowest n value, which could be attributed to greater entanglement or hydrogen bonding between CNFs and gelatin chains (Table 1).

In electrospinning, rheological behavior plays a crucial role, and the shear-thinning ability of a polymer solution can be attributed to the alignment of polymer chains and the predominance of the opening rate of chain entanglements over their formation rate [32]. Shear-thinning fluids are preferred over Newtonian fluids for electrospinning because they can improve the electrospinning performance and the nanofiber quality. Shear-thinning fluids have several advantages: for example, (1) they can form thinner and more uniform fibers, because the viscosity decreases as the jet is stretched by the electric field, reducing the jet diameter and facilitating fiber solidification; (2) they can produce a higher fiber yield with lower energy consumption, because when the apparent viscosity decreases, the flow rate increases, which enhances the material throughput and reduces the heat generation and solvent evaporation; (3) they can improve the spinnability and stability of the jet, because a decrease in the viscosity causes an increase in the electric field, preventing the jet from breaking up into droplets or beads [33].

The properties of fish skin gelatin in acetic acid/formic acid aqueous solutions were investigated by Songchotikunpan et al. (2008). They found that the concentration of the gelatin solutions had significant effects on their rheological and physical properties due to the effect on chain entanglement [34]. According to a study conducted by Wang et al. (2019), the incorporation of cellulose nanocrystals (CNCs) increased both the density and the number of hydrogen-bonding interactions between CNCs and the chitosan/poly (ethylene oxide) matrix, which, in turn, increased the viscosity [35].

#### 3.1.2. Electrical Conductivity Measurement

**Figure 2:**
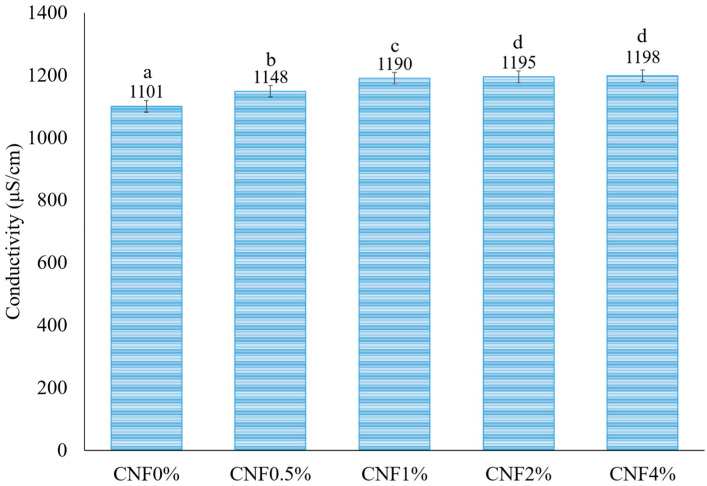
The conductivity of gelatin/CNF solutions containing different concentrations of CNFs. Error bars show the standard deviations of mean values (*n* = 3). Different letters on the columns indicate significant differences (*p* < 0.05) among the different values.

The conductivity of the polymer solution or melt determines how well the solution or melt can be charged and stretched (jet behavior after ejection from the tip) in the electric field, which, in turn, influences the spinnability (fiber formation) and quality of the nanofibers (morphology and mechanical properties) [36]. The conductivity data of different gelatin (15% *w*/*v* dissolved in a mixture of water/ethanol/acetic acid (3:2:3, *v*/*v*))–CNF solutions are shown in Figure 2. The concentration of CNFs (0–4%) had a relatively small effect on electrical conductivity. The electrical conductivity value of the gelatin solution was 1101 ± 0.7 μS/cm and increased to 1198 ± 2.12 μS/cm with the addition of 4% CNFs. Cellulose nanofibers are not ionized molecules, which means that they do not have charges by themselves [37]. However, impurities, such as metal ions, salts, or organic acids, can be present in the cellulose nanofibers due to the source of the cellulose, the extraction method, or the processing conditions [38]. These impurities can dissociate into ions or donate or accept electrons, which can increase the conductivity of the solution. For example, a study by Balea et al. (2019) showed that the conductivity of cellulose nanofiber solutions increased with increasing sodium hydroxide concentration, which was used as a solvent and a catalyst for cellulose extraction. The sodium hydroxide can dissociate into sodium and hydroxide ions, which can increase the charge density and the mobility of the solution [39]. Another example is a study by Jiao et al. (2021), who showed that the conductivity of cellulose nanofiber solutions increased with increasing sulfuric acid concentration, which was used as a hydrolyzing agent for cellulose extraction. Sulfuric acid can donate protons or accept electrons, which can increase the charge transfer and the redox reactions of the solution [40]. In the literature, similar increasing trends of conductivity were reported with increasing cellulose nanocrystal (CNC) content in poly (lactic acid) (PLA) solutions because of the uronic acid and sulfate ester groups on the cellulose nanocrystal surface [41]. Also, Ni et al. (2019) reported the same results when adding CNCs to electrospun poly (methyl methacrylate) nanofibers [42]. In another study, the results showed that the addition of CNCs to the chitosan–poly (ethylene oxide) spinning solution led to an increase in its conductivity [35].

The conductivity should be high enough to allow the jet to be charged, stretched, and retain its stability (preventing the jet from breaking up into droplets) in the electric field (spinnability); on the other hand, it is important for the formation of thin fibers without faulty morphologies and lower energy consumption aspects [43]. The low conductivity of a polymer solution/melt leads to a lack of jet stream formation or insufficient elongation, which may result in bead formation [33]. On the other hand, too high a conductivity causes (1) the excessive heating or evaporation of the solvent, which can cause the bending instability or irregularity of the jet, which can result in non-uniform or defective nanofibers with variability in the fiber diameter distribution, and (2) electrical breakdown or spark discharge, which can damage the equipment or the nanofibers [44,45,46]. The solution conductivity of electrospinning solutions is dependent on the solvent type, polymer type, and presence of ions in the solution. The presence of free ions is necessary for conductivity, as, without them, there is no mechanism for transporting electrical charge, and the electrical conductivity is zero [43]. When selecting the solvent(s) to dissolve the polymer, one of the solvents must have some degree of conductivity. Hence, alcohols and pure water, which do not ionize, are poor conductors of electricity [33]. The presence of acids, bases, and salts contributes to increased conductivity. In the current research, acetic acid was used in a mixed solvent (water, ethanol, and acid acetic) for increased conductivity.

#### 3.1.3. Surface Tension Measurement

The surface tension of a liquid is a cohesive force between molecules at the surface of a liquid that causes the liquid surface to behave as a thin elastic membrane [47]. The surface tension depends on the intermolecular forces between the molecules of the liquid and the surrounding medium.

**Figure 3:**
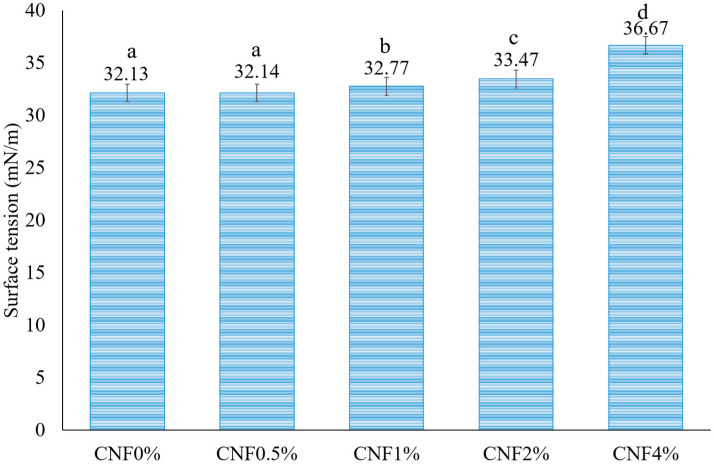
The surface tension of gelatin/CNF solutions containing different concentrations of CNFs. Error bars show the standard deviations of mean values (*n* = 3). Different letters on the columns indicate significant differences (*p* < 0.05) among the different values.

As presented in Figure 3, the surface tension of the gelatin (15% *w*/*v* dissolved in a mixture of water/ethanol/acetic acid (3:2:3, *v*/*v*))–CNF solution increased from 32.13 to 36.67 mN/m when the CNF concentration increased from 0 to 4%. When CNFs are added to a liquid, they form a network of fibrils that can interact with the liquid molecules and modify the surface tension. The CNFs create a more rigid and ordered structure at the liquid–air interface, which reduces the entropy and increases the free energy of the system [48]. The CNFs also reduce the mobility and flexibility of the liquid molecules, which increases the cohesive force between them [49]. Therefore, the liquid behaves as if it has a stronger elastic membrane at its surface. One possible reason for the decrease in spinnability is the increase in surface tension as the concentration of CNFs rises. The surface tension of a polymer solution is an important parameter in the electrospinning process because it affects both the formation and quality of the nanofibers. Electrospinning involves a charged solution to overcome surface tension, with lower surface tension facilitating electrospinning in a lower electric field [50]. The process aims to minimize the surface area per unit mass by forming spheres, while the electrical charges on the jet work to increase the surface area through elongation. As the solution jet accelerates, it stretches, but the surface tension can cause it to break into droplets [47]. On the other hand, lower surface tension can reduce the jet diameter and facilitate fiber solidification, resulting in thinner and more uniform fibers. However, if the surface tension is too low, it can cause instability or beading of the jet, leading to non-uniform or defective fibers [51]. According to previous research works, a spinning solution with a surface tension of 35–55 dyn/cm^2^ is considered to be suitable for electrospinning [52]. However, this value may vary depending on the type and composition of the polymer solution and the desired properties and applications of the nanofibers. The surface tension of the solution can be modified by mixing solvents. The surface tensions of ethanol (γ = ~22.39 mN/m) and acetic acid (γ = ~27.60 mN/m) are lower than that of water (γ = ~72.86 mN/m). So, in this research, a mixed solvent was used in electrospinning. Another method for decreasing the surface tension of an electrospinning solution is to use surfactants [52]. According to Aida et al. (2010), reducing the surface tension and increasing the conductivity of the solution can lead to the production of smoother fibers with smaller diameters, as it enhances the solution’s stretching capability [53]. Ki et al. (2005) found that when gelatin was dissolved at a low concentration in formic acid, the solution had low viscosity but relatively high surface tension, which meant that the jet stream lacked the ability to maintain its shape and broke down into small drops [54]. In a study conducted by Kriegel et al. (2009), the surface tension of chitosan and neutral poly(ethylene oxide) (PEO) solutions was investigated in different concentrations of acetic acid. The results showed that as the concentration of acetic acid increased from 50% to 90%, the surface tension decreased. Additionally, the addition of surfactants also caused a decrease in surface tension. Specifically, when anionic sodium dodecyl sulfate (SDS) was added to a 50% acetic acid solution, a significant decrease occurred in surface tension [31].

### 3.2. Determination of Optimal Gelatin Concentration in Electrospinning

In electrospinning, both the fiber formation (spinnability) and fiber quality (morphology, diameter, and mechanical properties) are strongly affected by the solution concentration. At low concentrations, the charged jets can lose their intermolecular attractions and separate into droplets, leading to electrospraying instead of electrospinning [5]. At high concentrations, significant entanglement can occur, resulting in a mixture of bead and fiber formation. To produce electrospun fibers, the polymer concentration should be at least 2–2.5 times the critical chain entanglement concentration (Ce) [55]. However, at very high concentrations, the viscosity becomes too high, weakening the electrical charge and potentially clogging the syringe [5]. It also leads to rapid solidification of the jet stream and reduces the stretching and thinning of the jet, resulting in thicker and less uniform fibers [44]. Optimal conditions for smooth, thin fibers are achieved when the polymer solution is moderately concentrated [56]. The optimal concentration depends on the type of polymer and solvent, the presence of additives, and electrospinning conditions. Therefore, it is advisable to perform a trial-and-error method to find the best concentration for a specific biopolymer and electrospinning setup for producing high-quality nanofibers with the desired properties.

In the current work, first, three gelatin solutions with different concentrations (10, 15, 20% *w*/*v*) were prepared in the mixed solvent (water/ethanol/acetic acid mixture at 3:2:3, *v*/*v*). The gelatin solutions at 15% *w*/*v* were successfully electrospun at room temperature, and smooth and continuous fibers were obtained. At the lowest concentration (10%), electrospraying instead of electrospinning was observed, and drops of the solution instead of fiber mats could be seen on the surface of the aluminum collector. At the highest solution concentration (20%), after starting electrospinning, highly viscous fluid balls were gradually gathered outside the needle tip, and a few fiber mats were deposited on the collector. So, the solution with a 15% concentration was selected as the optimal concentration for adding CNFs (0.5, 1, 2, and 4% *w*/*v*). There was no problem with the spinnability of solutions containing 0.5, 1, or 2% CNFs; however, there was difficulty in spinnability at 4% CNFs, which might be related to the viscosity of the solution being too high.

In some situations, despite the presence of a high concentration of biopolymer in the solvent, the solution cannot be electrospun due to low molecular weight, a wide molecular weight distribution, or high repulsive forces between the biopolymer chains, which prevents sufficient entanglement [5]. Increasing conductivity, decreasing the surface tension of solvents, using a second polymer, and changing equipment parameters may help to lower the concentration of the polymer required for fiber formation and increase its electrospinnability [47].

### 3.3. Morphological Study

**Figure 4:**
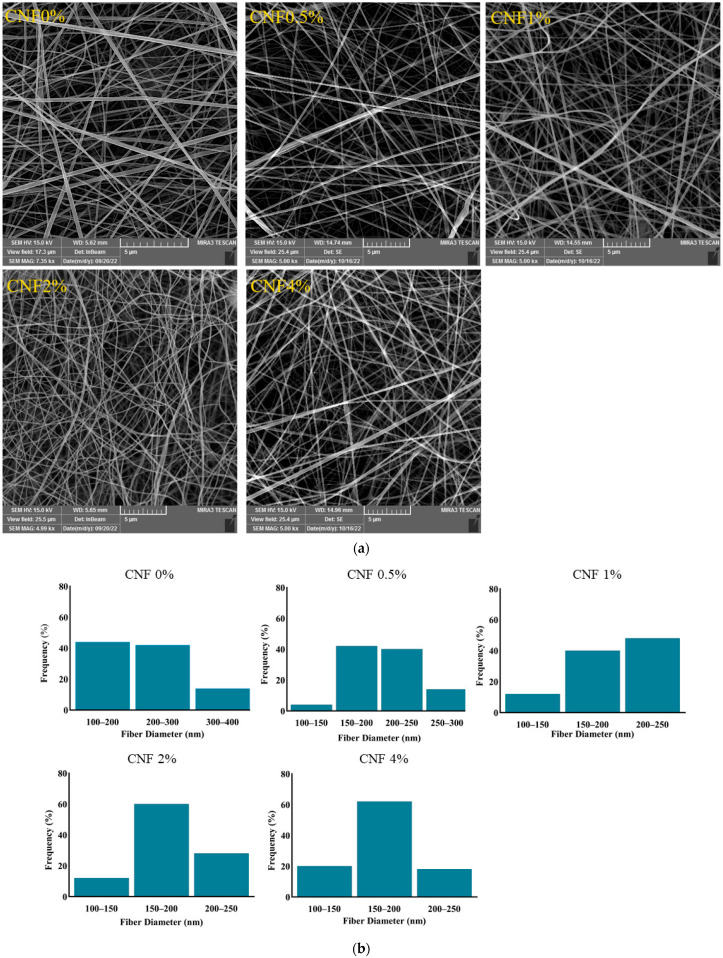
(**a**) FE-SEM images of gelatin/CNF hybrid nanofibers; (**b**) diameter distribution diagrams.

FE-SEM stands for field-emission scanning electron microscopy, which is a technique that can produce high-resolution images of the surface and morphology of nanomaterials. It can provide valuable information about the effects of electrospinning parameters on the microstructure and quality properties of nanofibers [57,58]. The resulting data help to optimize the electrospinning process and improve the quality and performance of nanofiber-based products [59,60]. FE-SEM was used to study the morphology of gelatin nanofibers with different CNF concentrations. Figure 4a,b show FE-SEM images and the diameter distribution of different nanofiber samples, demonstrating uniformly ordered network structures with good continuity and a fine diameter distribution, without fracture or bead formation. After analyzing the dimensions of the nanofibers, it can be found that the diameter of the gelatin/CNF nanofibers decreased with increasing CNF content, and the average diameter of the fibers decreased from 216 nm in the pure gelatin nanofibers to 175.39 nm in the gelatin/CNF (4%) nanofibers (Table 2). Adding cellulose nanofibers (CNFs) to a gelatin solution can decrease the diameter of the electrospun nanofibers, probably by increasing the entanglement, viscosity, and conductivity of the solution. These effects can enhance the stability and stretching of the jet stream, leading to thinner and more uniform fibers [61]. The presence of CNFs can promote better alignment of polymer chains. This alignment can contribute to the formation of finer fibers as the solution jet undergoes less entanglement and can be drawn into thinner strands. On the other hand, the addition of CNFs to the polymer solution can increase the viscosity. Higher viscosity can lead to thinner fibers because the electrospinning jet is more stable and can be stretched further before solidifying. The optimal diameter ranges of electrospun nanofiber for packaging and encapsulation purposes may depend on the type of material, the encapsulation efficiency, the release rate, and the stability of the encapsulated compounds [33]. Generally, smaller-diameter nanofibers can provide a higher surface area, porosity, and mechanical strength, which can improve the performance of the packaging and encapsulation materials [62]. However, too small a diameter may also cause some drawbacks, such as low permeability, high brittleness, and high cost. Therefore, the optimal diameter range should be determined by balancing the advantages and disadvantages of different sizes. According to some studies, the diameter ranges of electrospun nanofibers for packaging and encapsulation purposes can vary from tens of nanometers to several micrometers, depending on the application and the material [63].

### 3.4. FTIR Analysis

**Figure 5:**
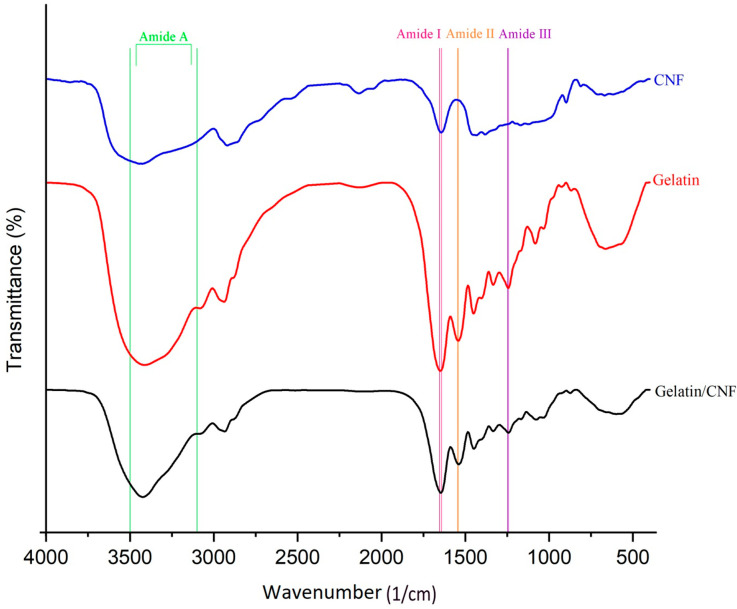
The FTIR spectra of CNFs, gelatin nanofiber, and gelatin/CNF hybrid nanofibers.

The FTIR spectra of the electrospun gelatin, gelatin/CNF nanofibers, and CNFs are illustrated in Figure 5. In detail, the characteristic peaks were interpreted as follows: The amide A band, which occurs at a wavelength range of 3100–3500 1/cm, is associated with the stretching of N-H bonds in primary amino groups found in proteins. Alternatively, it can be attributed to the stretching of O-H bonds in water molecules that are adsorbed onto the fibers [64]. Other notable bands include the stretching of C-H bonds at 3071–3076 1/cm, the stretching vibrations of C-H bonds in aliphatic groups at 2931–2959 1/cm, the stretching of C=O bonds at 1641–1653 1/cm (referred to as amide I), the bending and stretching of C-N bonds at 1541–1544 1/cm (referred to as amide II), and the combination band of N-H bend and C-N stretching. Additionally, there is the symmetric deformation of NH_3_^+^ at 1454 1/cm, the stretching of -C-N bonds at 1406–1412 1/cm, the deformation of C-H bonds in methyl groups at 1336 1/cm, and the stretching of C-N bonds and bending of N-H at 1242–1247 1/cm (amide III) [65,66,67].

Figure 5 displays the FTIR spectrum illustrating the impact of CNFs (4% incorporation) on the functional groups of gelatin in gelatin nanofibers. The incorporation of CNFs causes a shift to lower wavenumbers in the N-H amide peak as a result of overlapping CNF hydroxyl groups, O-H, in the gelatin/CNF 4% sample. Moreover, there is a discernible distinction in the spectra below 1200 1/cm due to overlapping CNF peaks with gelatin. Consequently, the FTIR analysis confirms the presence of a cellulose structure in the nanofibers.

### 3.5. Thermal Analysis

**Figure 6:**
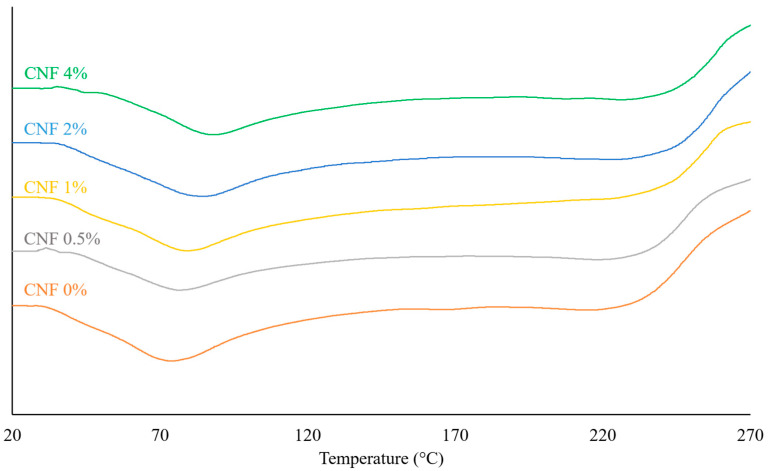
The DSC thermogram of gelatin/CNF hybrid nanofibers containing different concentrations of CNFs.

Differential scanning calorimetry (DSC) is a technique that can measure the heat flow associated with physical and chemical transitions in materials. In the current research, the effect of different CNF concentrations on the thermal properties of the hybrid electrospun gelatin/CNF nanofibers was investigated, and the thermograms are shown in Figure 6. At all DSC curves, endothermic peaks could be seen in the temperature range of 70–90 °C, which could be attributed to the glass transition temperature [8,68]. T_g_ is the temperature at which the polymer changes from a rigid and brittle state to a soft and rubbery state [69]. The T_g_ of biopolymers generally increases with decreasing moisture content and increasing cross-linking and crystallinity [70]. The pure gelatin-based electrospun fibers showed a T_g_ of 73.8 °C; adding CNFs caused an increase in T_g_, and an increase in CNF concentration led to an increase in the T_g_ of the hybrid nanofibers (Table 3). The formation of intermolecular hydrogen bonds between CNFs and gelatin may lead to the decreased mobility of gelatin chains, contributing to the observed results. These findings suggest that CNFs and gelatin are effectively miscible in the fiber matrices, resulting in an increased T_g_ [68]. The T_g_ of hybrid and composite polymers is affected by the extent of the interaction between polymer chains and other constituents, such as the filler and second polymer [71]. Similar behavior has been observed in the case of polyvinyl alcohol (PVA) reinforced by cellulose nanocrystals [72].

The thermograms of pure gelatin fibers did not show a first-order endothermic transition peak (melting/denaturation) for gelatin, indicating that the structure of gelatin had turned from semi-crystalline to an amorphous state. Also, the effective denaturation of the gelatin protein due to heating, shearing, the electrical field, and the organic solvent in electrospinning probably caused the disappearance of corresponding endothermic denaturation peaks in the DSC thermogram of the resulting nanofibers [8,73]. The melting temperature (T_m_) of a protein is the temperature at which the crystalline regions of the protein melt; however, not all proteins have crystalline regions, and some proteins are mostly amorphous and do not show a clear melting transition [74]. On the other hand, proteins usually show a sharp endothermic peak (T_d_) when they undergo denaturation (for example, at ~105–120 °C for pure gelatin), which is the irreversible loss of their native structure and function [75]. This peak corresponds to the heat absorbed by the protein as it unfolds and exposes its hydrophobic regions to the solvent. Some proteins may show more than one peak in DSC, indicating that they have multiple domains or subunits that denature at different temperatures [74].

DSC can be used to study the electrospun fibers from various aspects, such as (1) determining the thermal properties of the fibers, such as the glass transition temperature (T_g_), melting temperature (T_m_), crystallization temperature (T_c_), and heat capacity (C_p_); (2) evaluating the thermal stability and degradation temperature (T_de_) of the fibers by performing thermal gravimetric analysis (TGA) coupled with DSC; (3) investigating the effects of electrospinning parameters on the crystallinity of biopolymers used in nanofibers, and (4) determining the encapsulated compound in electrospun fibers by analyzing the thermal behavior of the fibers and comparing it with that of the pure components [63,76,77]. The decomposition temperature (T_de_) is the temperature at which the biopolymer undergoes thermal degradation or pyrolysis. This transition is usually observed as both exo- and endothermic phenomena in the DSC curve [78]. According to previous reports, the thermal degradation of gelatin starts with an endothermic peak at temperatures above 230 °C, and then exothermic peaks appear at temperatures above 280 °C [74]. In this research, the starting of thermal decomposition was observed at ~220 °C, and the degradation temperature was increased by adding and increasing the concentration of CNFs. Other researchers also noted increased thermal stability in polymers combined with CNFs [68,79]. It is necessary to mention that, depending on whether the aluminum pan of DSC equipment is sealed or open, which determines the evaporation or non-evaporation of water, the number, location, and magnitude of endothermic peaks will be different in the DSC thermograms of biopolymers. The evaporation of water can cause the appearance of a large endothermic peak in the temperature range of 70–125 °C that overlaps with small peaks, such as those corresponding to melting, denaturation, and glass transition [74].

### 3.6. XRD Analysis

**Figure 7:**
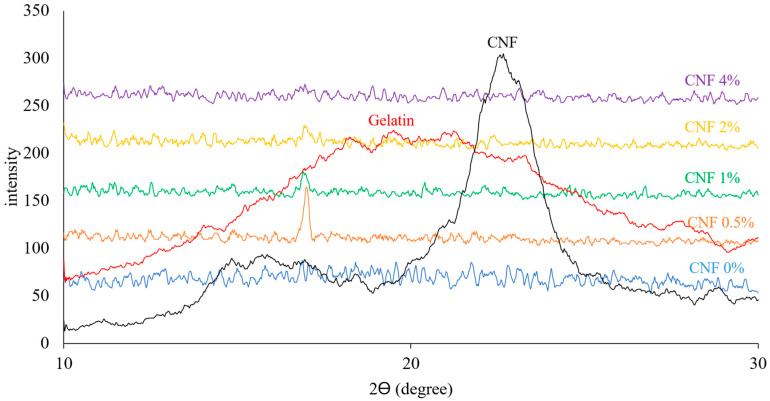
X-ray diffraction (XRD) patterns of gelatin powder, gelatin/CNF hybrid nanofibers, and CNFs.

Figure 7 shows the XRD diffractograms of CNFs, gelatin powder, and gelatin/CNF hybrid nanofibers. The peaks at the angles of 2θ = 14.84°, 15.84°, 16.94°, and 22.66° in the CNF diffractogram are related to the structure of cellulose I [80]. These peaks correspond to the (101), (101), (002), and (040) crystallographic planes. High peak intensity and resolution indicate high purity and crystallization levels of CNFs [17]. Gelatin powder exhibits the typical XRD pattern of gelatin’s semi-crystalline structure originating from a triple-helical structure. In the gelatin/CNF hybrid nanofibers, more amorphous structures were formed; however, there was a narrow peak at 16.9° corresponding to an interplanar spacing of 5.22 Å [2].

XRD analysis can be used in the study of electrospun fibers for different purposes, including (1) determining the crystallinity and crystallite size in the nanofibers, which can affect their mechanical, optical, and electrical properties; (2) investigating additives’ effects (second polymer, improver compounds, encapsulated bioactive materials) on the crystalline state of hybrid nanofibers; (3) studying the compatibility and miscibility of these additives with the major polymer; and (4) evaluating the effects of the electrospinning process and variables (such as voltage, flow rate, concentration, and solvent) on the crystallinity of nanofibers [81,82,83]. Crystallinity is the degree and arrangement of order in the molecular structure of a polymer and is an important factor that affects the water solubility and mechanical, thermal, and optical properties of electrospun nanofibers. For example, higher crystallinity can improve the stiffness, strength, water barrier property, and thermal stability of nanofibers but lower their ductility and transparency [84,85].

Biopolymer-based electrospun nanofibers can have either crystalline or amorphous structures, depending on the type of biopolymer, the electrospinning parameters, and the post-treatment methods. Generally, proteins tend to form more semi-crystalline nanofibers, while polysaccharides tend to form more amorphous nanofibers [5]. However, there are exceptions and variations, such as the effect of the solvent, concentration, voltage, temperature, humidity, and cross-linking on the crystallinity and morphology of the nanofibers [86]. According to previous research, gelatin powder usually shows a semi-crystalline structure with a triple-helix conformation, while electrospun gelatin nanofibers appear to have a more amorphous structure with a random coil conformation [68,87]. When biopolymers are solubilized in solvents and subjected to the electrospinning process, the bonds between polymer chains can be disrupted, leading to an increase in the amorphous structure. On the other hand, electrospinning rapidly stretches and solidifies the molecular chains of polymers, leading to the formation of an amorphous structure and preventing crystallization [88]. However, some studies have also reported the formation of crystalline nanofibers from gelatin by using certain solvents, such as acetic acid, or by applying post-treatment methods, such as annealing or glutaraldehyde cross-linking [87,89].

The previous literature has also demonstrated that gelatin does not recrystallize from a formic acid solution, resulting in mostly amorphous gelatin nanofibers with little crystallinity [54].

### 3.7. Mechanical Property Measurements

**Figure 8:**
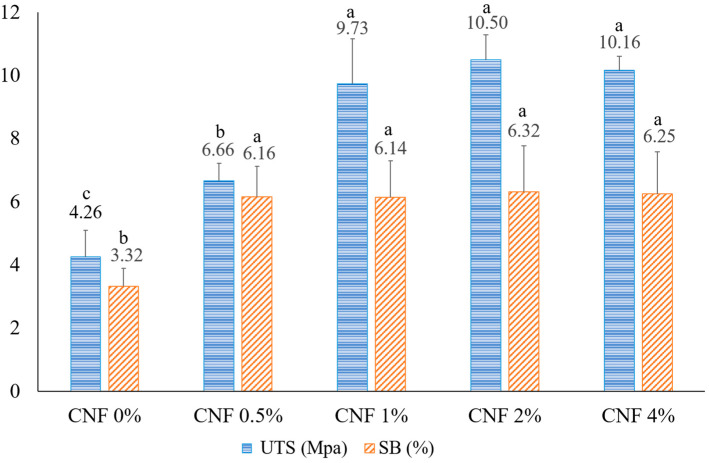
Tensile properties of gelatin/CNF hybrid nanofiber mats with 0.01 mm thickness. Error bars show the standard deviations of mean values (*n* = 3). Different letters indicate significant differences (*p* < 0.05) among the different values.

The mechanical properties of the gelatin/CNF-based hybrid nanofiber films containing varying concentrations of CNFs (0, 0.5, 1, 2, and 4%) were evaluated by tensile testing. The effects of the CNF concentration on the ultimate tensile strength (UTS) and strain at break (SB) are shown in Figure 8. The UTS and SB of nanofiber films increased from 4.26 to 10.5 MPa and 3.3% to 6.25% when adding 2% CNFs, respectively, while the average diameter of the gelatin/CNF hybrid nanofiber mats was lower than that of the pure gelatin ones. The increase in the UTS and SB of nanofiber films can be attributed to the following mechanisms: (1) the FTIR results indicate the formation of new hydrogen bonds between gelatin and CNFs; (2) additionally, the long length of CNFs contributes to the formation of a network-like structure and reduces the mobility of the polymeric chains; (3) moreover, CNFs fill the free volume between gelatin chains, playing a crucial role as a nanofiller and further decreasing chain mobility; and (4) there is a mechanical percolation effect between CNFs and gelatin chains [33,61]. In this phenomenon, the addition of a low concentration of CNFs to a biopolymer matrix can significantly improve the mechanical properties of the composite, such as its stiffness, strength, and toughness, because CNFs can form a stiff, interconnected, continuous network of nanofibers that act as load-bearing and stress-transfer elements from the soft gelatin matrix to the stiff CNFs to resist the tensile process, as well as restrict the mobility and deformation of the biopolymer chains. The mechanical percolation effect depends on the dispersion, orientation, and interaction of CNFs with the biopolymer matrix [90].

When the CNF concentration increased to 4%, a non-significant decrease was observed, which could be attributed to the aggregation of CNFs at high levels in electrospun fibers, especially on the fiber surface, which could weaken the cohesion between fibers. Also, a thinner nanofiber mat was formed at 4% CNF due to the high viscosity of the solution and the lack of ejection of a sufficient jet stream (more time was needed to reach the same thickness of the mat). Shi et al. (2012) reported similar results when increasing the cellulose nanocrystal (CNC) content in PLA nanofiber films [41].

## 4. Conclusions

Different concentrations of CNFs (0.5 to 4%) were used to improve the morphology and mechanical and physical properties of gelatin-based nanofibers. Gelatin solutions (15% *w*/*v*) containing CNFs were prepared in a mixed solvent (water/ethanol/acetic acid), and then hybrid electrospun nanofibers were produced. The rheological properties of the solutions, including the viscosity and shear-thinning degree, were incrementally affected by the CNF concentration, which could be attributed to an increase in chain entanglement. CNFs also increased the surface tension, which may be related to solvent–polymer interactions. An increase in viscosity and shear-thinning degree is desirable for electrospinning; however, an increase in surface tension is undesirable. On the other hand, CNFs had little effect on solution conductivity, probably because the cellulose chains are not ionized. However, impurities, such as metal ions, salts, or organic acids, can be present in cellulose nanofibers, which can slightly affect conductivity. The diameter of the gelatin/CNF hybrid nanofibers decreased with increasing CNF content, and all hybrid nanofibers had a bead-free and smooth morphology, showing that CNFs had positive effects on the morphology of electrospun gelatin nanofibers. The mechanical properties of hybrid nanofiber mats/films were also improved by CNFs at 2%, while the average diameter of the gelatin/CNF hybrid nanofiber mats was lower than that of the pure gelatin ones. CNFs also increased the T_g_ of electrospun nanofibers but did not affect their crystallinity. This research shows that CNFs have a high ability to improve electrospun mats/films, which can potentially increase the application range of these types of nanofibers. However, more investigations on hybrids between CNFs and other biopolymer-based electrospun nanofibers are required for the development of their application for industrial purposes.

## Figures and Tables

**Table 1:** The apparent viscosity and power-law model parameters of different gelatin/CNF solutions.

Solutions	k (Pa·s ^n^)	n	Apparent Viscosity (mPa·s) at 10 1/s
CNF 0%	0.38 ± 0.04	0.83 ± 0.01	200.34 ± 6.20
CNF 0.5%	0.41 ± 0.06	0.83 ± 0.01	219.4 ± 8.31
CNF 1%	0.57 ± 0.02	0.86 ± 0.02	347.08 ± 4.72
CNF 2%	0.68 ± 0.08	0.7 ± 0.01	220.68 ± 7.11
CNF 4%	1.7 ± 0.1	0.51 ± 0.03	279.79 ± 6.76

k: consistency index; n: flow behavior index.

**Table 2:** The average diameter and quality of the gelatin/CNF hybrid nanofibers.

	CNF 0%	CNF 0.5%	CNF 1%	CNF 2%	CNF 4%
Number of nanofibers	50	50	50	50	50
Average diameter	216	205.67	198.85	182.16	175.39
Standard deviation	56.6	39.17	33.31	27.65	31.22
Min (nm)	132	106.33	122.54	117.56	109.4
Max (nm)	329	286.3	265.11	243.61	272.59
Quality	Bead-free fibers	Bead-free fibers	Bead-free fibers	Bead-free fibers	Bead-free fibers

**Table 3:** The glass transition temperature of gelatin/CNF hybrid nanofibers.

**CNF Content in Gelatin Nanofibers**	**Glass Transition Temperature (T_g_ °C)**
0%	73.8 ± 1.2
0.5%	76.1 ± 2.1
1%	79 ± 1.1
2%	83 ± 2.3
4%	87.5 ± 1.3

## Data Availability

The original contributions presented in the study are included in the article, further inquiries can be directed to the corresponding authors.

## References

[1] Aghaei Z., Ghorani B., Emadzadeh B., Kadkhodaee R., Tucker N. (2020). Protein-based halochromic electrospun nanosensor for monitoring trout fish freshness. Food Control.

[2] Deng L., Zhang X., Li Y., Que F., Kang X., Liu Y., Feng F., Zhang H. (2018). Characterization of gelatin/zein nanofibers by hybrid electrospinning. Food Hydrocoll..

[3] Guo M., Wang H., Wang Q., Chen M., Li L., Li X., Jiang S. (2020). Intelligent double-layer fiber mats with high colorimetric response sensitivity for food freshness monitoring and preservation. Food Hydrocoll..

[4] Ardestani S.A., Ghanbarzadeh B., Moini S. (2023). The improvement of the sodium caseinate based electrospun nanofiber by modifying solvent system: Study of microstructure and physical properties. Food Hydrocoll..

[5] Wang Y., Khan M.A., Chen K., Zhang L., Chen X. (2023). Electrospinning of natural biopolymers for innovative food applications: A review. Food Bioprocess Technol..

[6] Jahangir M.U., Wong S.Y., Afrin H., Nurunnabi M., Li X., Arafat M.T. (2022). Understanding the solubility and electrospinnability of gelatin using Teas approach in single/binary organic solvent systems. Bull. Mater. Sci..

[7] Khan M.R., Sadiq M.B. (2021). Importance of gelatin, nanoparticles and their interactions in the formulation of biodegradable composite films: A review. Polym. Bull..

[8] Sharma G.K., Jalaja K., Ramya P., James N.R. (2022). Electrospun Gelatin Nanofibres—Fabrication, Cross-linking and Biomedical Applications: A Review. Biomed. Mater. Devices.

[9] Nowzari F., Shábanpour B., Ojagh S.M. (2013). Comparison of chitosan–gelatin composite and bilayer coating and film effect on the quality of refrigerated rainbow trout. Food Chem..

[10] Ramos M., Valdés A., Beltrán A., Garrigós M.C. (2016). Gelatin-based films and coatings for food packaging applications. Coatings.

[11] Mahmood K., Kamilah H., Sudesh K., Karim A.A., Ariffin F. (2019). Study of electrospun fish gelatin nanofilms from benign organic acids as solvents. Food Packag. Shelf Life.

[12] Cetinkaya T., Bildik F., Altay F., Ceylan Z. (2024). Gelatin nanofibers with black elderberry, Au nanoparticles and SnO_2_ as intelligent packaging layer used for monitoring freshness of Hake fish. Food Chem..

[13] Lin L., Gu Y., Cui H. (2018). Novel electrospun gelatin-glycerin-ε-Poly-lysine nanofibers for controlling Listeria monocytogenes on beef. Food Packag. Shelf Life.

[14] Zhou Y., Miao X., Lan X., Luo J., Luo T., Zhong Z., Gao X., Mafang Z., Ji J., Wang H. (2020). Angelica essential oil loaded electrospun gelatin nanofibers for active food packaging application. Polymers.

[15] Jancikova S., Jamróz E., Kulawik P., Tkaczewska J., Dordevic D. (2019). Furcellaran/gelatin hydrolysate/rosemary extract composite films as active and intelligent packaging materials. Int. J. Biol. Macromol..

[16] Dufresne A. (2006). Comparing the mechanical properties of high performances polymer nanocomposites from biological sources. J. Nanosci. Nanotechnol..

[17] Savadekar N.R., Karande V.S., Vigneshwaran N., Bharimalla A.K., Mhaske S.T. (2012). Preparation of nano cellulose fibers and its application in kappa-carrageenan based film. Int. J. Biol. Macromol..

[18] Rhim J.W., Park H.M., Ha C.S. (2013). Bio-nanocomposites for food packaging applications. Prog. Polym. Sci..

[19] Jensen A., Lim L.T., Barbut S., Marcone M. (2015). Development and characterization of soy protein films incorporated with cellulose fibers using a hot surface casting technique. LWT.

[20] Almasi H., Ghanbarzadeh B., Dehghannya J., Entezami A.A., Asl A.K. (2015). Novel nanocomposites based on fatty acid modified cellulose nanofibers/poly (lactic acid): Morphological and physical properties. Food Packag. Shelf Life.

[21] Deepa B., Abraham E., Pothan L.A., Cordeiro N., Faria M., Thomas S. (2016). Biodegradable nanocomposite films based on sodium alginate and cellulose nanofibrils. Materials.

[22] Oun A.A., Rhim J.W. (2015). Preparation and characterization of sodium carboxymethyl cellulose/cotton linter cellulose nanofibril composite films. Carbohydr. Polym..

[23] Shankar S., Rhim J.W. (2016). Preparation of nanocellulose from micro-crystalline cellulose: The effect on the performance and properties of agar-based composite films. Carbohydr. Polym..

[24] Kaushik A., Singh M., Verma G. (2010). Green nanocomposites based on thermoplastic starch and steam exploded cellulose nanofibrils from wheat straw. Carbohydr. Polym..

[25] Ibrahim M.M., El-Zawawy W.K., Nassar M.A. (2010). Synthesis and characterization of polyvinyl alcohol/nanospherical cellulose particle films. Carbohydr. Polym..

[26] Cherian B.M., Leão A.L., de Souza S.F., Costa L.M.M., de Olyveira G.M., Kottaisamy M., Nagarajan E., Thomas S. (2011). Cellulose nanocomposites with nanofibres isolated from pineapple leaf fibers for medical applications. Carbohydr. Polym..

[27] Petroudy S.R.D., Garmaroody E.R., Rudi H. (2017). Oriented cellulose nanopaper (OCNP) based on bagasse cellulose nanofibrils. Carbohydr. Polym..

[28] (1991). Standard Test Method for Tensile Properties of Thin Plastic Sheeting.

[29] Valipour P., Ghasemi S. (2017). Effect of non-Newtonian rheology on electrified jets of polymer nanofibers in electrospinning process based on bead–spring model. Int. J. Adv. Manuf. Technol..

[30] Nayak P., Ghosh A.K., Bhatnagar N. (2022). Investigation of solution rheology in electrospinning of ultra-high molecular weight polyethylene. Fiber Polym..

[31] Kriegel C., Kit K., McClements D.J., Weiss J. (2009). Electrospinning of chitosan–poly (ethylene oxide) blend nanofibers in the presence of micellar surfactant solutions. Polymer.

[32] Zhang X., Tang K., Zheng X. (2015). Electrospinning and rheological behavior of poly (vinyl alcohol)/collagen blended solutions. J. Wuhan Univ. Technol.-Mater. Sci. Ed..

[33] Tahir M., Vicini S., Sionkowska A. (2023). Electrospun Materials Based on Polymer and Biopolymer Blends—A Review. Polymers.

[34] Songchotikunpan P., Tattiyakul J., Supaphol P. (2008). Extraction and electrospinning of gelatin from fish skin. Int. J. Biol. Macromol..

[35] Wang D., Cheng W., Wang Q., Zang J., Zhang Y., Han G. (2019). Preparation of electrospun chitosan/poly (ethylene oxide) composite nanofibers reinforced with cellulose nanocrystals: Structure, morphology, and mechanical behavior. Compos. Sci. Technol..

[36] Aydogdu A., Sumnu G., Sahin S. (2018). A novel electrospun hydroxypropyl methylcellulose/polyethylene oxide blend nanofibers: Morphology and physicochemical properties. Carbohydr. Polym..

[37] Rahmani S., Khoubi-Arani Z., Mohammadzadeh-Komuleh S., Maroufkhani M. (2021). Electrospinning of cellulose nanofibers for advanced applications. Handbook of Nanocelluloses: Classification, Properties, Fabrication, and Emerging Applications.

[38] Zhang Y., Zhang C., Wang Y. (2021). Recent progress in cellulose-based electrospun nanofibers as multifunctional materials. Nanoscale Adv..

[39] Balea A., Sanchez-Salvador J.L., Monte M.C., Merayo N., Negro C., Blanco A. (2019). In situ production and application of cellulose nanofibers to improve recycled paper production. Molecules.

[40] Jiao Y., Lu Y., Lu K., Yue Y., Xu X., Xiao H., Li J., Han J. (2021). Highly stretchable and self-healing cellulose nanofiber-mediated conductive hydrogel towards strain sensing application. J. Colloid Interface Sci..

[41] Shi Q., Zhou C., Yue Y., Guo W., Wu Y., Wu Q. (2012). Mechanical properties and in vitro degradation of electrospun bio-nanocomposite mats from PLA and cellulose nanocrystals. Carbohydr. Polym..

[42] Ni X., Cheng W., Huan S., Wang D., Han G. (2019). Electrospun cellulose nanocrystals/poly (methyl methacrylate) composite nanofibers: Morphology, thermal and mechanical properties. Carbohydr. Polym..

[43] Mirtič J., Balažic H., Zupančič Š. (2019). Effect of solution composition variables on electrospun alginate nanofibers: Response surface analysis. Polymers.

[44] SalehHudin H.S., Mohamad E.N., Mahadi W.N.L., Muhammad Afifi A. (2018). Multiple-jet electrospinning methods for nanofiber processing: A review. Mater. Manuf. Process..

[45] Vega-Lugo A.C., Lim L.T. (2012). Effects of poly (ethylene oxide) and pH on the electrospinning of whey protein isolate. J. Polym. Sci. Part B Polym. Phys..

[46] Wilk S., Benko A. (2021). Advances in fabricating the electrospun biopolymer-based biomaterials. J. Funct. Biomater..

[47] Sharma G.K., James N.R. (2022). Electrospinning: The Technique and Applications. Recent Developments in Nanofibers Research.

[48] Zhang K., Barhoum A., Xiaoqing C., Li H., Samyn P. (2019). Cellulose nanofibers: Fabrication and surface functionalization techniques. Handbook of Nanofibers.

[49] Meftahi A., Momeni Heravi M.E., Baroum A., Samyn P., Najarzadeh H., Alibakhshi S., Barhoum A. (2020). Cellulose Nanofibers. Handbook of Nanocelluloses: Classification, Properties, Fabrication, and Emerging Applications.

[50] Andrady A.L. (2008). Science and Technology of Polymer Nanofibers.

[51] Bhardwaj N., Kundu S.C. (2010). Electrospinning: A fascinating fiber fabrication technique. Biotechnol. Adv..

[52] Jia L., Qin X.H. (2013). The effect of different surfactants on the electrospinning poly (vinyl alcohol) (PVA) nanofibers. J. Therm. Anal. Calorim..

[53] Aida T.M., Yamagata T., Watanabe M., Smith R.L. (2010). Depolymerization of sodium alginate under hydrothermal conditions. Carbohydr. Polym..

[54] Ki C.S., Baek D.H., Gang K.D., Lee K.H., Um I.C., Park Y.H. (2005). Characterization of gelatin nanofiber prepared from gelatin–formic acid solution. Polymer.

[55] Lu Y., Li Y., Zhang S., Xu G., Fu K., Lee H., Zhang X. (2013). Parameter study and characterization for polyacrylonitrile nanofibers fabricated via centrifugal spinning process. Eur. Polym. J..

[56] Rezaeinia H., Ghorani B., Emadzadeh B., Tucker N. (2019). Electrohydrodynamic atomization of Balangu (*Lallemantia royleana*) seed gum for the fast-release of *Mentha longifolia* L. essential oil: Characterization of nano-capsules and modeling the kinetics of release. Food Hydrocoll..

[57] Langwald S.V., Ehrmann A., Sabantina L. (2023). Measuring Physical Properties of Electrospun Nanofiber Mats for Different Biomedical Applications. Membranes.

[58] Mamun A., Klöcker M., Blachowicz T., Sabantina L. (2022). Investigation of the Morphological Structure of Needle-Free Electrospun Magnetic Nanofiber Mats. Magnetochemistry.

[59] Das A., Balakrishnan N.T., Joyner J.D., Medhavi N., Manaf O., Jabeen Fatima M., Ahn J.H., Ali W., Prasanth R. (2021). Electrospinning: The state of art technique for the production of nanofibers and nanofibrous membranes for advanced engineering applications. Electrospinning for Advanced Energy Storage Applications. Materials Horizons: From Nature to Nanomaterials.

[60] Nadaf A., Gupta A., Hasan N., Ahmad S., Kesharwani P., Ahmad F.J. (2022). Recent update on electrospinning and electrospun nanofibers: Current trends and their applications. RSC Adv..

[61] Chinnappan B.A., Krishnaswamy M., Xu H., Hoque M.E. (2022). Electrospinning of biomedical nanofibers/nanomembranes: Effects of process parameters. Polymers.

[62] Liu S., White K.L., Reneker D.H. (2019). Electrospinning polymer nanofibers with controlled diameters. IEEE Trans. Ind. Appl..

[63] Wen P., Wen Y., Zong M.H., Linhardt R.J., Wu H. (2017). Encapsulation of bioactive compound in electrospun fibers and its potential application. J. Agric. Food Chem..

[64] Kutzli I., Gibis M., Baier S.K., Weiss J. (2019). Electrospinning of whey and soy protein mixed with maltodextrin–Influence of protein type and ratio on the production and morphology of fibers. Food Hydrocoll..

[65] Muyonga J., Cole C., Duodu K. (2004). Fourier transform infrared (FTIR) spectroscopic study of acid soluble collagen and gelatin from skins and bones of young and adult Nile perch (*Lates niloticus*). Food Chem..

[66] Nguyen T.H., Lee B.T. (2010). Fabrication and characterization of cross-linked gelatin electro-spun nano-fibers. J. Biomed. Sci. Eng..

[67] Siimon K., Reemann P., Poder A., Pook M., Kangur T., Kingo K., Jaks V., Mäeorg U., Jaervekuelg M. (2014). Effect of glucose content on thermally cross-linked fibrous gelatin scaffolds for tissue engineering. Mater. Sci. Eng. C.

[68] Barkane A., Kampe E., Platnieks O., Gaidukovs S. (2021). Cellulose nanocrystals vs. cellulose nanofibers: A comparative study of reinforcing effects in uv-cured vegetable oil nanocomposites. Nanomaterials.

[69] Mazur K., Singh R., Friedrich R.P., Genç H., Unterweger H., Sałasińska K., Bogucki R., Kuciel S., Cicha I. (2020). The effect of antibacterial particle incorporation on the mechanical properties, biodegradability, and biocompatibility of PLA and PHBV composites. Macromol. Mater. Eng..

[70] Jouki M., Yazdi F.T., Mortazavi S.A., Koocheki A. (2014). Quince seed mucilage films incorporated with oregano essential oil: Physical, thermal, barrier, antioxidant and antibacterial properties. Food Hydrocoll..

[71] Kropka J.M., Putz K.W., Pryamitsyn V., Ganesan V., Green P.F. (2007). Origin of dynamical properties in PMMA−C60 nanocomposites. Macromolecules.

[72] George J., Ramana K., Bawa A. (2011). Bacterial cellulose nanocrystals exhibiting high thermal stability and their polymer nanocomposites. Int. J. Biol. Macromol..

[73] Kotatha D., Hirata M., Ogino M., Uchida S., Ishikawa M., Furuike T., Tamura H. (2019). Preparation and characterization of electrospun gelatin nanofibers for use as nonaqueous electrolyte in electric double-layer capacitor. J. Nanotechnol..

[74] Mukherjee I., Rosolen M. (2013). Thermal transitions of gelatin evaluated using DSC sample pans of various seal integrities. J. Therm. Anal. Calorim..

[75] Tseretely G., Smirnova O. (1992). DSC study of melting and glass transition in gelatins. J. Therm. Anal. Calorim..

[76] Mirjalili M., Zohoori S. (2016). Review for application of electrospinning and electrospun nanofibers technology in textile industry. J. Nanostructure Chem..

[77] Nikmaram N., Roohinejad S., Hashemi S., Koubaa M., Barba F.J., Abbaspourrad A., Greiner R. (2017). Emulsion-based systems for fabrication of electrospun nanofibers: Food, pharmaceutical and biomedical applications. RSC Adv..

[78] Szcześniak L., Rachocki A., Tritt-Goc J. (2008). Glass transition temperature and thermal decomposition of cellulose powder. Cellulose.

[79] Abdulkhani A., Hosseinzadeh J., Ashori A., Dadashi S., Takzare Z. (2014). Preparation and characterization of modified cellulose nanofibers reinforced polylactic acid nanocomposite. Polym. Test..

[80] Trovatti E., Fernandes S.C., Rubatat L., da Silva Perez D., Freire C.S., Silvestre A.J., Neto C.P. (2012). Pullulan–nanofibrillated cellulose composite films with improved thermal and mechanical properties. Compos. Sci. Technol..

[81] Bkkar M., Olekhnovich R., Kremleva A., Sitnikova V., Kovach Y., Zverkov N., Uspenskaya M. (2023). Influence of electrospinning setup parameters on properties of polymer-perovskite nanofibers. Polymers.

[82] Islam M.S., Rahaman M.S., Yeum J.H. (2015). Electrospun novel super-absorbent based on polysaccharide–polyvinyl alcohol–montmorillonite clay nanocomposites. Carbohydr. Polym..

[83] Zhu G., Sheng L., Tong Q. (2014). Preparation and characterization of carboxymethyl-gellan and pullulan blend films. Food Hydrocoll..

[84] Huang F., Wei Q., Wang J., Cai Y., Huang Y. (2008). Effect of temperature on structure, morphology and crystallinity of PVDF nanofibers via electrospinning. e-Polymers.

[85] Lim C., Tan E., Ng S. (2008). Effects of crystalline morphology on the tensile properties of electrospun polymer nanofibers. Appl. Phys. Lett..

[86] Ramzan A., Intisar A., Mumtaz N., Hussain N., Bilal M. (2022). Applications of Biopolymer-Based Nanofibers and Nanomembranes. Handbook of Biopolymers.

[87] Chi H.Y., Chang N.Y., Li C., Chan V., Hsieh J.H., Tsai Y.H., Lin T. (2022). Fabrication of gelatin nanofibers by electrospinning—Mixture of gelatin and polyvinyl alcohol. Polymers.

[88] Jalili M., Mozaffari A., Gashti M., Parsania M. (2019). Electrospinning Nanofibers Gelatin scaffolds: Nanoanalysis of properties and optimizing the process for tissue engineering functional. J. Nanoanalysis.

[89] Aytac Z., Sen H.S., Durgun E., Uyar T. (2015). Sulfisoxazole/cyclodextrin inclusion complex incorporated in electrospun hydroxypropyl cellulose nanofibers as drug delivery system. Colloids Surf. B Biointerfaces.

[90] Muthukrishnan L. (2022). An overview on electrospinning and its advancement toward hard and soft tissue engineering applications. Colloid Polym. Sci..

